# TiO_2_ Phase Junction Electron Transport Layer Boosts Efficiency of Planar Perovskite Solar Cells

**DOI:** 10.1002/advs.201700614

**Published:** 2018-01-06

**Authors:** Yayun Zhu, Kaimo Deng, Haoxuan Sun, Bangkai Gu, Hao Lu, Fengren Cao, Jie Xiong, Liang Li

**Affiliations:** ^1^ College of Physics, Optoelectronics and Energy Jiangsu Key Laboratory of Thin Films Center for Energy Conversion Materials & Physics (CECMP) Soochow University Suzhou 215006 P. R. China; ^2^ State Key Laboratory of Electronic Thin Films and Integrated Devices University of Electronic Science and Technology of China Chengdu 610054 P. R. China

**Keywords:** atomic layer deposition, electron transport layers, phase junctions, perovskites, titanium oxide

## Abstract

In the planar perovskite solar cells (PSCs), the electron transport layer (ETL) plays a critical role in electron extraction and transport. Widely utilized TiO_2_ ETLs suffer from the low conductivity and high surface defect density. To address these problems, for the first time, two types of ETLs based on TiO_2_ phase junction are designed and fabricated distributed in the opposite space, namely anatase/rutile and rutile/anatase. The champion efficiency of PSCs based on phase junction ETL is over 15%, which is much higher than that of cells with single anatase (9.8%) and rutile (11.8%) TiO_2_ as ETL. The phase junction based PSCs also demonstrated obviously reduced hysteresis. The enhanced performance is discussed and mainly ascribed to the excellent capability of carrier extraction, defect passivation, and reduced recombination at the ETL/perovskite interface. This work opens a new phase junction ETL strategy toward interfacial energy band manipulation for improved PSC performance.

## 1 Introduction

Organic–inorganic lead halide perovskites have attracted increasing attention as light absorbers of solar cells. Since the first report in 2009, the power conversion efficiencies (PCEs) of perovskite solar cells (PSCs) have increased rapidly from 3.8% to over 22.1%.^1^, ^2^, ^3^, ^4^, ^5^, ^6^ Perovskite solar cells have good prospect due to their advantage, such as low‐cost technology, high light absorption, long carrier diffusion length, high carrier mobility, and engineered energy band.^7^, ^8^, ^9^, ^10^ PSCs have different device architecture including mesoscopic, planar, and inverted structure.^11^, ^12^, ^13^ No matter what kind of cells, the uniform coverage of perovskite and perfect interface properties is crucial to device performance.^14^ Traditional PSCs consist of transparent anode layer, electron transport layer (ETL), light absorption layer, hole transport layer (HTL), and metal electrode (Ag or Au). As for planar PSCs, ETL plays an important role in carrier extraction and transport, which suppresses the recombination of electrons with holes generated in the perovskite.^15^, ^16^, ^17^ Until now, TiO_2_ is still widely considered to be the preferred ETL for PSCs because of its matched conduction band (CB) with perovskite, and thus the high ability of electron injection and collection is obtained. However, the application of TiO_2_ ETL in PSCs is also limited owing to some disadvantage such as low conductivity and a large amount of defects, leading to the unavoidable carrier recombination.^18^, ^19^

To improve the efficiency of PSCs, much efforts have been made to resolve the above concern for TiO_2_ ETLs. Doping, nanocomposites, and interface engineering are the most effective strategies for improving the performance of TiO_2_ based PSCs.^20^, ^21^, ^22^, ^23^, ^24^, ^25^, ^26^, ^27^ For example, metal ions (Mg, Nb, Li, Y, and so on) doped TiO_2_ have been utilized as efficient ETLs to enhance the efficiency to over 19%, resulting from improved capability of carrier extraction or injection.^20^, ^21^, ^22^, ^23^ The incorporation of highly conductive carbon materials, such as carbon dots, graphene, and carbon nanotubes to form nanocomposites has been proved to be an efficient method for increasing the electron mobility and reducing the photocurrent hysteresis.^24^, ^25^, ^26^ Recently, we introduced a CdS layer at the TiO_2_/perovskite interface to passivate surface defects and increase Fermi level in planar PSCs, effectively suppressing the recombination and raising open voltages of cells.^27^ Heterojunctions with a built‐in potential have been frequently employed in optoelectronic conversion devices including solar cells; however, it is scarce to investigate heterojunction as ETL for boosting the efficiency of PSCs.

Among different types of heterojunctions, novel phase junctions constructed by one semiconductor with two phases have been demonstrated to be effective in separating electron–hole pairs at the interface, which provides a new route to manipulate charge transport of semiconductor devices, particularly in photoelectrochemical cells.^28^, ^29^, ^30^ Here, we design and synthesize TiO_2_ phase junction as ETL in planar PSCs, which are obtained by combining two phase of TiO_2_ in the opposite space, namely anatase/rutile (AR) and rutile/anatase (RA). To the best of our knowledge, this is the first report of TiO_2_ phase junction based PSCs. The phase junctions passivate defects and enhance charge transport property at the ETL/perovskite interface, leading to improved efficiency and reduced hysteresis. The PCEs of champion devices with AR and RA structure are 15.11% and 15.33%, respectively, which are significantly higher than those of reference cells with the single anatase (9.8%) and rutile TiO_2_ (11.8%) as ETL.

## 2 Results and Discussion

**Figure 1:**
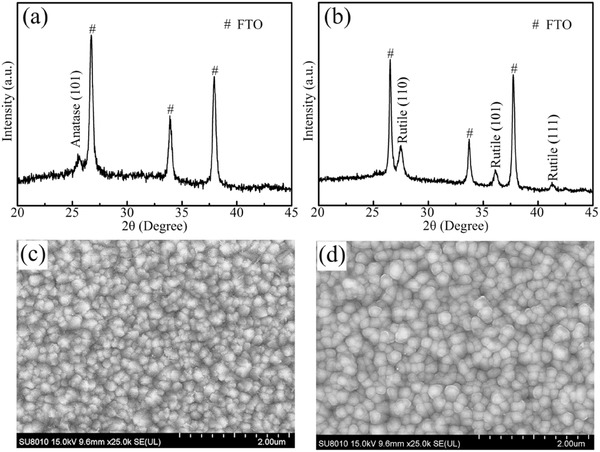
XRD pattern of TiO_2_ layers synthesized by a) ALD and b) water bath reaction. SEM images of c) AR and d) RA TiO_2_ phase junction films on FTO substrates.

**Figure** **1**a,b shows the X‐ray diffraction (XRD) pattern of TiO_2_ layers on fluorine‐doped tin oxide (FTO) substrates fabricated by atomic layer deposition (ALD) and water bath reaction in the TiCl_4_ solution, respectively. For the ALD TiO_2_ based substrate, the film thickness is about 10 nm and a weak diffraction peak appears at 26°, which is ascribed to the (101) plane of anatase phase TiO_2_ (JCPDS 21–1272). Raman spectra in Figure S1a (Supporting Information) further prove the formation of anatase TiO_2_. The XRD pattern (Figure 1b) of film synthesized in the water bath demonstrates the characteristic of rutile TiO_2_ (JCPDS 21–1276). The above results indicate that the anatase and rutile TiO_2_ films can be fabricated by ALD and TiCl_4_ solution reaction, respectively. Consequently, we can fabricate anatase/rutile (simplified as AR) and rutile/anatase (simplified as RA) phase junction films on FTO substrates by adjusting the deposition order of ALD and water bath reaction. As shown in Figure S2 (Supporting Information), the diffraction peaks corresponding to the rutile TiO_2_ phase and anatase TiO_2_ phase appear in the XRD pattern, which confirms the existence of phase junction. The top‐view scanning electron microscopy (SEM) images of FTO with AR and RA films are given in Figure 1c,d, indicating the TiO_2_ layers are evenly coated on the FTO surface compared with the SEM image of bare FTO shown in Figure S1b (Supporting Information). SEM images of the single layered films are shown in Figure S1c,d (Supporting Information). The morphology of the layers indicates no obvious difference, which ensures these two ETLs will have negligible effects on the formation of perovskite films. Both the AR and RA TiO_2_ coated FTO substrates remain transparent (Figure S1e, Supporting Information) and the transmittance of AR structure is almost identical with bare FTO substrate (Figure S1f, Supporting Information), which is beneficial for device performance due to the great utilization of incident light. As for RA films, the values of transmission decline obviously and the utilization rate of incident light is lower than AR films, but the higher device performance is found in the RA structure. This phenomenon implies superior electronic transport in the RA structure, which will be discussed in the next section.

**Figure 2:**
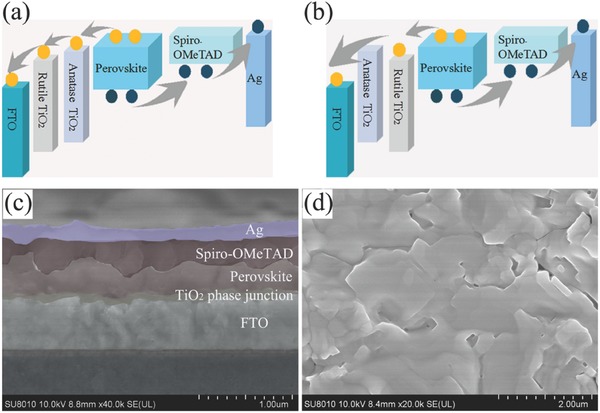
Schematic representation of perovskite solar cells with a) RA and b) AR phase junction as ETL. c) Cross‐sectional SEM image of RA ETL based PSC. d) Top‐view SEM image of perovskite film on the RA‐25 nm TiO_2_/FTO substrates.

The ultraviolet photoemission spectroscopy (UPS) measurements were performed to reveal the relative position of conduction band and valence band (VB) of anatase and rutile TiO_2_, as shown in Figure S3 (Supporting Information). The corresponding curves of enlarged parts are presented in Figure S4 (Supporting Information). The secondary electron onset (S.O.) on the left side of the spectra is 16.07 and 16.22 eV for anatase and rutile TiO_2_ (Figure S4a,c, Supporting Information), respectively. The work function of anatase TiO_2_ and rutile TiO_2_ is calculated to be 5.13 and 4.98 eV by subtracting the S.O. position from the excitation energy (21.2 eV) of the He I UPS spectra. The value of valence band maximum of anatase and rutile TiO_2_ is located at 2.88 and 3.10 eV (Figure S4b,d, Supporting Information), respectively, determined by making a straight line into the leading edge. According to the absorption spectrum (Figure S5a, Supporting Information), the band gap of anatase and rutile TiO_2_ is 3.3 and 3.23 eV, respectively. Combining with Figure S4 (Supporting Information), we can determine the CB and VB energy levels of TiO_2_ films (Figure S5b, Supporting Information). Based on the above results, **Figure** **2**a,b (Supporting Information) shows the energy band diagram of different parts in two types of PSCs. The relative energy band position of anatase and rutile TiO_2_ is consistent with previous reports. It clearly indicates that the conduction band of rutile TiO_2_ is slightly lower than that of anatase TiO_2_. The RA structure favors the electron transport from perovskite to TiO_2_ layer, while anatase TiO_2_ serves as a barrier layer in the AR structure to hinder the electron recombination with holes in perovskites. A typical cross‐section SEM image of RA TiO_2_ ETL based PSC is presented in Figure 2c. The top‐view SEM image of perovskite film on RA TiO_2_ is shown in Figure 2d. It can be seen that the perovskite layer is pinhole free and the crystal size is on the order of micrometer, both of which are favorable for light absorption and photocurrent generation. The SEM image of perovskite on the AR TiO_2_ is also given in Figure S6 (Supporting Information). No obvious difference is found for the perovskite layer, indicating that the bottom TiO_2_ layer has negligible effect on the morphology of perovskite.

**Figure 3:**
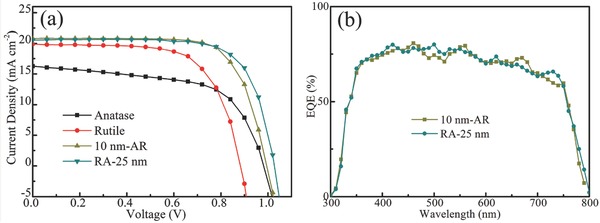
a) *J*–*V* curves of perovskite solar cells based on anatase, rutile, 10 nm‐AR, and RA‐25 nm TiO_2_ ETLs. b) EQE spectra of 10 nm‐AR and RA‐25 nm devices.

The photocurrent density–voltage (*J*–*V*) curves of champion devices based on different TiO_2_ structures are shown in **Figure** **3**a. The corresponding photovoltaic parameters are summarized in **Table** **1**, which are acquired on the basis of average data of randomly selected 15 cells for every device. *J*–*V* curves measured at different scan rates and waiting time are presented in Figure S7 (Supporting Information). It can be found that the values of *V*_oc_ are almost same, while the *J*_sc_ shows slight decrease with decreased scanning rates and increased waiting time. Compared to the single anatase and rutile TiO_2_ based devices, *V*_oc_, FF, and *J*_sc_ of the TiO_2_ phase junction based PSCs are greatly improved, and the RA and AR TiO_2_ based PSCs have the highest PCEs of 15.3% and 15.1%, respectively. In these two types of phase junctions, the experimental parameters for the rutile TiO_2_ deposition are kept the same and the optimized concentration of TiCl_4_ solution is 0.1 mol L^−1^ based on the *J*–*V* performance (Figure S8a, Supporting Information). From the cross‐sectional SEM image in Figure S9 (Supporting Information), the deposited rutile TiO_2_ layer has a thickness of about 60 nm. For the AR structure, the optimized thickness of anatase TiO_2_ is 10 nm and the performance of devices based on different thicknesses of anatase TiO_2_ is provided in Figure S8b (Supporting Information). For the RA structure, the device with the 25 nm thickness of anatase TiO_2_ has the best performance (Figure S8c, Supporting Information). Further increasing the thickness, the larger electron transport length and more recombination will exist, resulting in the decline of all the photovoltaic parameters. The detailed discussion will be presented in the next section. Figure 3b shows the external quantum efficiency (EQE) spectrum of RA‐25 nm and 10 nm‐AR devices and the integrated *J*_sc_ are 18.00 and 18.12 mA cm^−2^, respectively, which are slightly lower than the values of 20.53 and 20.82 mA cm^−2^ measured from *J*–*V* curves.

**Table 1:** Summary of critical physical parameters of solar cells with different types of ETLs

Devices	*V*_oc_ [V]	*J*_sc_ [mA cm^−2^]	FF [%]	PCE [%]
Anatase	0.98 ± 0.04	17.2 ± 0.15	60.00 ± 0.83	9.85 ± 0.03
Rutile	0.91 ± 0.04	19.85 ± 0.43	65.51 ± 0.71	11.83 ± 0.43
5 nm‐AR	0.93 ± 0.07	20.51 ± 0.17	66.40 ± 0.78	12.90 ± 0.52
10 nm‐AR	1.00 ± 0.01	20.82 ± 0.16	72.99 ± 0.88	15.11 ± 0.58
15 nm‐AR	0.96 ± 0.03	20.37 ± 0.28	66.92 ± 1.53	12.25 ± 0.52
RA‐10 nm	0.97 ± 0.02	20.78 ± 0.06	66.26 ± 0.94	13.33 ± 0.46
RA‐15 nm	0.97 ± 0.01	21.00 ± 0.32	68.66 ± 1.15	13.61 ± 0.37
RA‐20 nm	1.02 ± 0.02	20.68 ± 0.05	69.99 ± 0.37	14.50 ± 0.57
RA‐25 nm	1.02 ± 0.02	20.53 ± 0.15	72.66 ± 0.86	15.33 ± 0.58
RA‐30 nm	0.97 ± 0.02	20.38 ± 0.49	67.52 ± 0.67	13.32 ± 0.43

**Figure 4:**
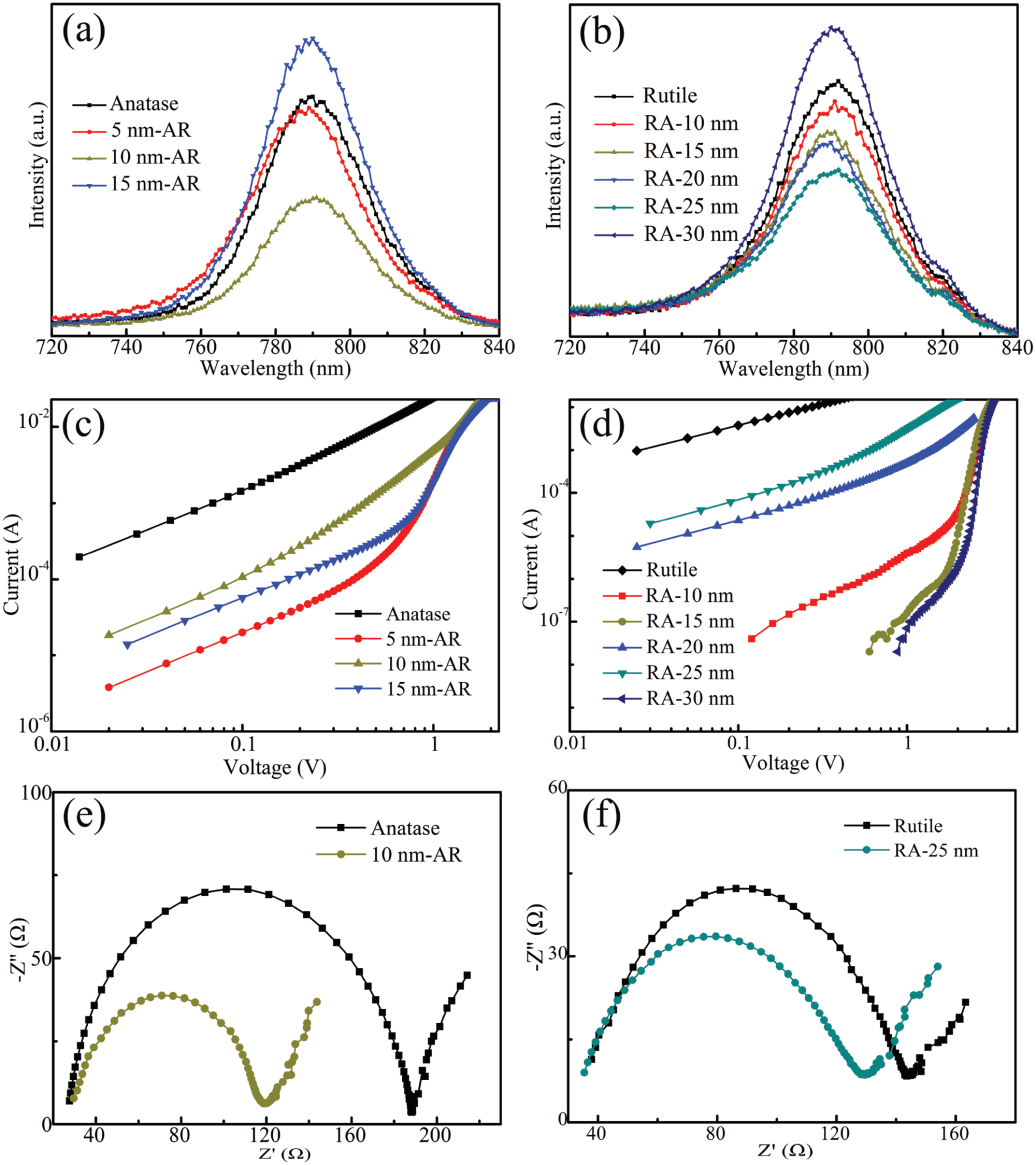
PL spectra of perovskite films deposited on a) anatase and AR, and b) rutile and RA phase junction TiO_2_ films. *I*–*V* curves of hole‐only devices fabricated on c) AR and d) RA films. Nyquist plots of PSCs with e) anatase and 10 nm‐AR, and f) rutile and RA‐25 nm TiO_2_ films as ETLs measured at 0 bias.

To understand the recombination behavior of carriers in our devices, we measured the room temperature photoluminescence (PL) emission spectra (**Figure** **4**a,b). The PL peaks of all the perovskite films deposited on different ETL layers locate at around 790 nm, corresponding to the band gap of perovskite. With increasing the thickness of anatase TiO_2_, the PL intensity of both AR and RA system first declines and then rises. The lower PL intensity indicates that the ETL layers effectively extract carriers from the perovskite and the number of carriers for the radiative recombination is reduced. From the PL emission spectra of AR phase junctions and anatase ETL based devices, we can observe a PL intensity decline when the thickness of anatase increases from 5 to 10 nm and an increase as the thickness further increases to 15 nm. This phenomenon suggests the 10 nm thick anatase TiO_2_ is an optimal parameter, which is consistent with the *J*–*V* results. As for the RA phase junction and rutile ETL, the PL intensity is the lowest when the thickness increases to 25 nm. PL results confirm that the charge transfer at the TiO_2_ phase junction ETL/perovskite interface is promoted compared with the single anatase and rutile phase.

The electron trap density in TiO_2_ films was evaluated by measuring *J*–*V* curves using the space charge limited current method in hole‐only diode (Au/TiO_2_/Au) devices in the dark (Figure S10, Supporting Information). As shown in Figure 4c,d, phase junction samples have the characteristic of Ohmic contact at a low bias voltage and the trap filled limit (TFL) transition point at a higher voltage. The voltage at the TFL transition point denoted as *V*_TFL_ is associated with the trap density (*N*_t_) according to the following equation^31^, ^32^

(1) $$V_{\mathrm{TFL}}=\frac{eN_{t}d^{2}}{2\varepsilon \varepsilon_{0}}$$

where *e* is the element charge amount, *d* is the length of electron to transport, ε is the dielectric constant, and ε_0_ is the permittivity of vacuum. The lower the *V*_TFL_ is, the lower the trap density is. With the optimal thickness of anatase TiO_2_, the *V*_TFL_ value is the lowest for both types of TiO_2_ phase junctions, indicating the trap states are effectively passivated under this condition.

Electrochemical impedance spectroscopy (EIS) was measured to investigate the charge transport and recombination dynamics in devices.^33^, ^34^ Figure 4e,f exhibits the Nyquist plots of AR, RA, anatase, and rutile champion devices under the AM 1.5G illumination at 0 V bias voltage. EIS data of other devices are provided in Figure S11a,b (Supporting Information). All the curves contain two circular arcs. The charge transfer resistance (*R*_ct_) at the ETL/perovskite interface can be fitted from the high‐frequency arc, while the lower‐frequency arc represents the charge recombination resistance (*R*_rec_) which is inversely proportional to recombination rate. According to the equivalent circuit model in Figure S11c (Supporting Information), the detailed fitted values of series resistance (*R*_s_), *R*_rec_, and *R*_ct_ are listed in Table S1 (Supporting Information). Compared with the single layer ETL based cells, both the AR and RA structures show the declined *R*_ct_ and increased *R*_rec_. In the 10 nm‐AR device, the *R*_ct_ is decreased to half of the value of the single anatase based device, demonstrating improved charge transfer capability at the AR/perovskite interface. Further increasing the thickness of ALD TiO_2_ layer will extend the transport distance, and thus carriers easily accumulate at the anatase/rutile phase interface, leading to a larger *R*_ct_. The increased value of *R*_rec_ indicates that carriers from FTO through AR ETL to recombine with holes in the perovskite are hindered. In RA devices, carriers would transfer from perovskite to ETL due to the type‐II band alignment between RA TiO_2_ and perovskite. All the values of *R*_rec_ have an obvious increase compared to single layer based devices. For the 25 nm thickness of ALD TiO_2_, the carrier recombination rate is the smallest and then increases again owing to the internal resistance in the thicker 30 nm TiO_2_ film. The *R*_ct_ of RA‐25 nm device is also the lowest resulting from the appropriate band alignment. Therefore, we can draw a conclusion that the 10 nm‐AR and RA‐25 nm devices show the lowest transfer resistance and the highest recombination resistance in comparison with the single anatase and rutile ETL based devices.

**Figure 5:**
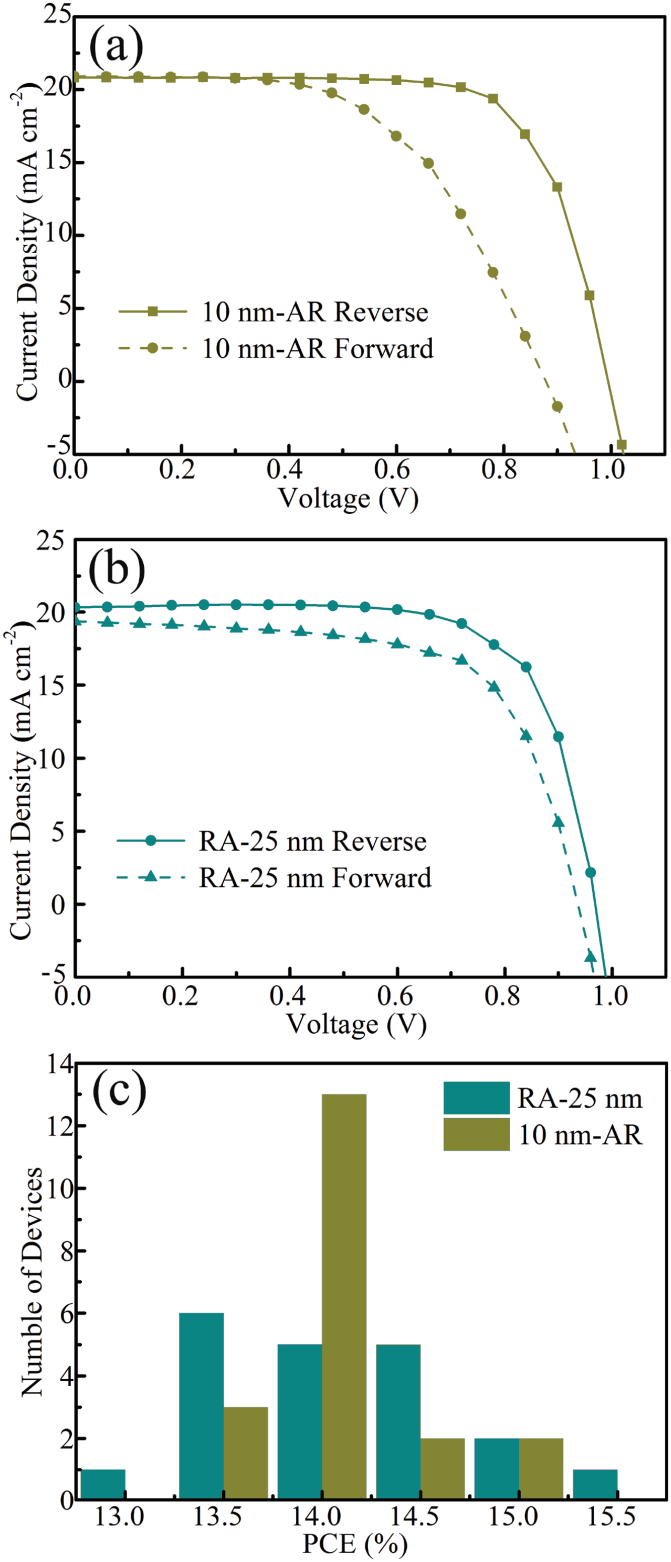
The *J*–*V* curves measured in the FS and RS direction. a) 10 nm‐AR, and b) RA‐25 PSC. c) Histogram of PCEs for 10 nm‐AR and RA‐25 nm PSCs obtained from 20 devices.

To reveal the stability of PSCs, the *J*–*V* curves of randomly selected 10 nm‐AR and RA‐25 nm based devices measured with the forward scan (FS) or reverse scan (RS) are given in **Figure** **5**a,b. Single anatase or rutile based devices were also measured and shown in Figure S12a,b (Supporting Information). The corresponding photovoltaic parameters are summarized in Table S2 (Supporting Information). For the 10 nm‐AR ETL based PSC, PCEs of 14.04% and 10.12% are obtained under the reverse and forward scan. Interestingly, RA‐25 nm based PSC has a reduced hysteresis with a PCE of 12.05% at the forward scan and 13.90% at the reverse scan. The lowered hysteresis in the RA device should be related with the more efficient charge transfer at the RA/perovskite interface, because the RA phase junction forms a type‐II structure and thus facilitates the carrier flow. The maximum power point tracking of the typical device with the AR and RA phase is provided in Figure S13 (Supporting Information), showing the devices can maintain a stable performance under the continued light illumination. We prepared a batch of 20 devices based on AR and RA ETLs and the PCE distribution is exhibited in Figure 5c. An average efficiency of 14.04% and 14.07% is obtained for the AR and RA ETL based devices, respectively, suggesting that the present devices have good reproducibility and stable performance.

## 3 Conclusions

In summary, TiO_2_ phase junctions with type‐II energy band structure have been successfully obtained by combing the ALD and water bath reaction. Employing the TiO_2_ phase junction as ETL, two types of planar perovskite cells with different phase order are fabricated. Compared with single phase TiO_2_ based ETL, TiO_2_ phase junction based PSCs demonstrate improved FF, *J*_sc_, and PCE with reduced hysteresis. The optimized rutile/anatase and anatase/rutile based devices yield the champion PCEs of 15.33% and 15.11%, respectively. The outstanding performance of TiO_2_ phase junction ETL based devices is mainly ascribed to the passivation of trap states, enhanced carrier extraction capability as well as reduced recombination rate. This work may open up new opportunities for fabricating high‐efficiency planar perovskite solar cells based on interfacial energy band control of ETLs.

## 4 Experimental Section

*Preparation of AR and RA ETLs on FTO Substrates*: The FTO substrates were patterned by etching with Zn powder and diluted HCl, and then cleaned by sequential ultrasonic treatment in acetone, alcohol, and deionized (DI) water each for 20 min. The AR ETL was grown on FTO substrate by ALD followed by a water bath reaction process. ALD anatase TiO_2_ with different thickness (5, 10, and 15 nm) was deposited on the FTO substrate and then immersed in the 0.1 m TiCl_4_ aqueous solution at 343 K for 60 min to obtain rutile TiO_2_. Meanwhile, the 10 nm ALD TiO_2_/FTO substrates without TiCl_4_ treatment served as the reference. The RA ETL was grown on FTO substrate by first immersing in the 0.1 m TiCl_4_ solution at 343 K for 60 min and then different thicknesses (10, 15, 20, 25, and 30 nm) of ALD TiO_2_ films were deposited. The TiCl_4_ treated FTO substrates without ALD were also prepared as reference. Finally, after washing with DI water, the substrates with TiO_2_ films were annealed at 773 K in air for 120 min.

*Fabrication of Perovskite Solar Cells*: The perovskite film was deposited on top of ETL by a modified one‐step method. The 1 mol L^−1^ perovskite solution was prepared by dissolving 231.8 mg PbCl_2_ (99.9985%, Alfa Aesar) and 397.7 mg CH_3_NH_3_ (99.5%, Alfa Aesar) in 1 mL of *N*,*N*‐dimethylformamide (DMF, 99.9%, Alfa Aesar), and stirring at 333 K for 12 h. Perovskite precursor was spin coated onto the ETL film at 3000 rpm for 40 s and then treated with chlorobenzene at 2000 rpm for 40 s. The perovskite coated ETL/FTO substrates were placed at room temperature for 30 min and then heated at 373 K for 50 min to remove the chlorobenzene and DMF solvent. After cooling down to room temperature, HTL solution was spin coated on the perovskite film at 2000 rpm for 30 s. The HTL solution was prepared by dissolving 72.3 mg of spiro‐OMeTAD, 28.8 µL of 4‐*tert*‐butyl pyridine, and 17.5 µL of lithium bis‐(trifluoromethanesulfonyl) imide (Li‐TFSI) (520 mg Li‐TFSI in 1 mL acetonitrile, 99.8%, Sigma‐Aldrich) in 1 mL of chlorobenzene (99.9%, Alfa Aesar). All the above procedures were conducted in the glove box with inert atmosphere. Finally, a 100 nm thick Ag electrode was deposited by thermal evaporation with a shadow mask (0.15 cm^2^ active area).

*Characterization*: The morphology of the samples was characterized by a field‐emission scanning electron microscope (Hitachi, SU8010). The energy band structure was evaluated by the UPS (Thermo Scientific, Escalab 250Xi). The phase of samples was measured using an XRD (D/MAX‐III‐B‐40KV, Cu Kα radiation, λ = 0.15418 nm). The PL spectrum was recorded by a spectrofluorometer (Horiba, Fluoromax‐4) with a 520 nm excitation wavelength at room temperature. The current density versus voltage (*J*–*V*) curves of all the devices were measured using a Newport solar simulator under AM1.5G irradiation (100 mW cm^−2^) with a Keithley 2400 Sourcemeter. The EQE curves were tested with a Newport QE 200 system. The absorption spectra were collected by a UV–vis spectrophotometer (Shimadzu, UV‐3600). The EIS was measured by an electrochemical workstation (Autolab, PGSTAT 302N) under illumination at 0 V bias voltages with an alternative signal amplitude of 5 mV and in the frequency range of 400–0.01 KHz.

## Conflict of Interest

The authors declare no conflict of interest.

## Supporting information

Supplementary
