# Supplementary material for: TiO2 Phase Junction Electron Transport Layer Boosts Efficiency of Planar Perovskite Solar Cells
Source: Adv Sci (Weinh). 2018 Jan 6;5(3):1700614. doi: 10.1002/advs.201700614 (PMC5867052; doi:10.1002/advs.201700614)
Supplement: Supplementary file 1 — Supplementary [file ADVS-5-1700614-s001.pdf]

Copyright WILEY-VCH Verlag GmbH & Co. KGaA, 69469 Weinheim, Germany, 2017.

ADVANCED SCIENCE
Open Access

Supporting Information

for *Adv. Sci.,* DOI: 10.1002/advs.201700614

TiO_2 Phase Junction Electron Transport Layer Boosts Efficiency of Planar Perovskite Solar Cells

Yayun Zhu, Kaimo Deng, Haoxuan Sun, Bangkai Gu, Hao Lu, Fengren Cao, Jie Xiong, and Liang Li**

Supporting Information

TiO_2 Phase Junction Electron Transport Layer Boosts Efficiency of Planar Perovskite Solar Cells

Yayun Zhu, Kaimo Deng, Haoxuan Sun, Bangkai Gu, Hao Lu, Fengren Cao, Jie Xiong, and Liang Li**

Y. Zhu, K. Deng, B. Gu, H. Lu, F. Cao, Prof. L. Li

College of Physics, Optoelectronics and Energy, Jiangsu Key Laboratory of Thin Films, Center for Energy Conversion Materials & Physics (CECMP), Soochow University, Suzhou 215006, P. R. China

Email: lli@suda.edu.cn

H. Sun, Prof. J. Xiong

State Key Laboratory of Electronic Thin Films and Integrated Devices, University of Electronic Science and Technology of China, Chengdu 610054, P. R. China

Email: jiexiong@uestc.edu.cn

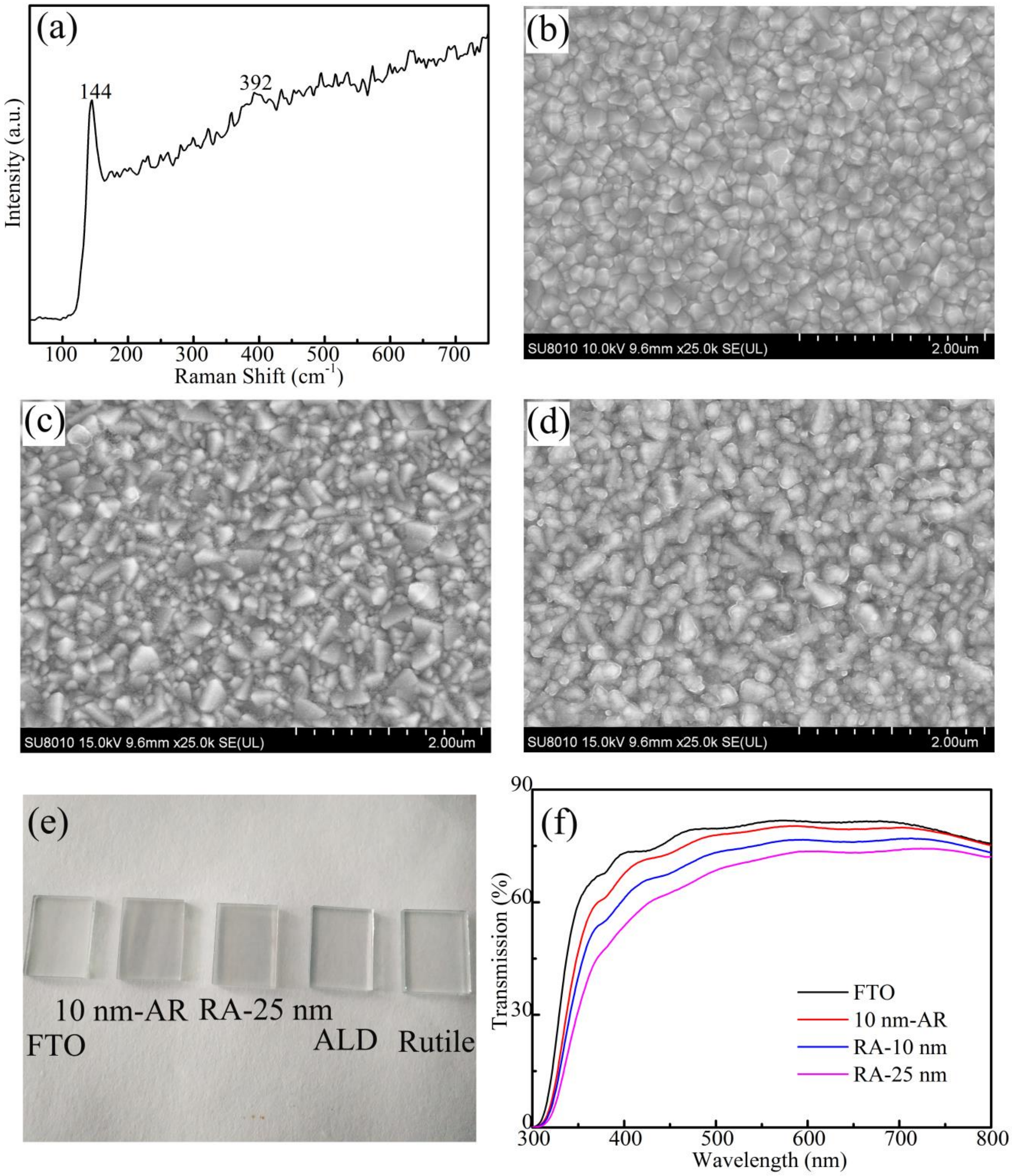

Figure S1. a) Raman spectrum of 10 nm thick ALD TiO_2 films. Top-view SEM of b) bare FTO, c) ALD 10 nm TiO_2/FTO, d) rutile TiO_2/FTO. e) The photos of FTO glass, 10 nm-AR TiO_2/FTO, RA-25 nm TiO_2/FTO, single ALD TiO_2/FTO, and single rutile TiO_2/FTO. f) Transmission spectrum of FTO, AR TiO_2/FTO, and RA TiO_2/FTO films.

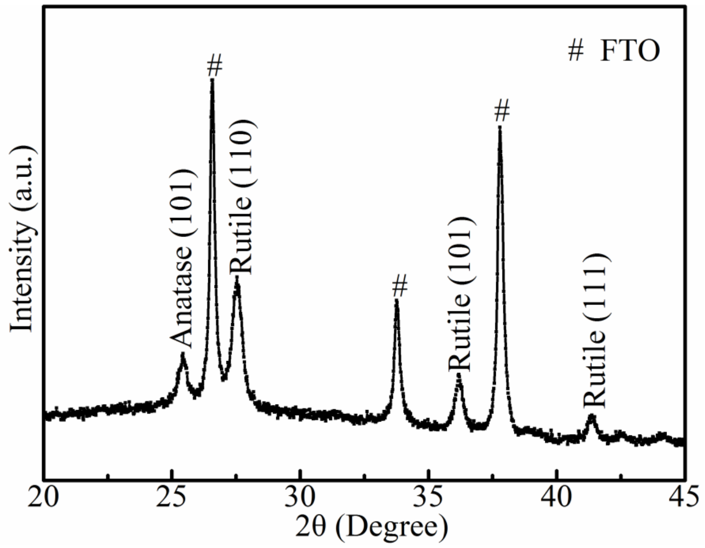

Figure S2. XRD pattern of TiO_2 RA phase junction films on FTO substrates.

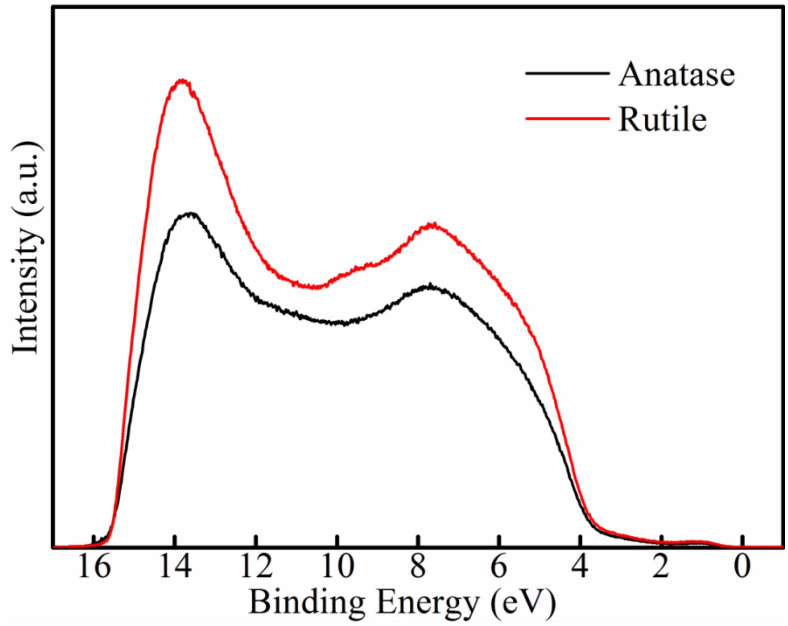

Figure S3. a) UPS spectra of anatase and rutile TiO_2 layers.

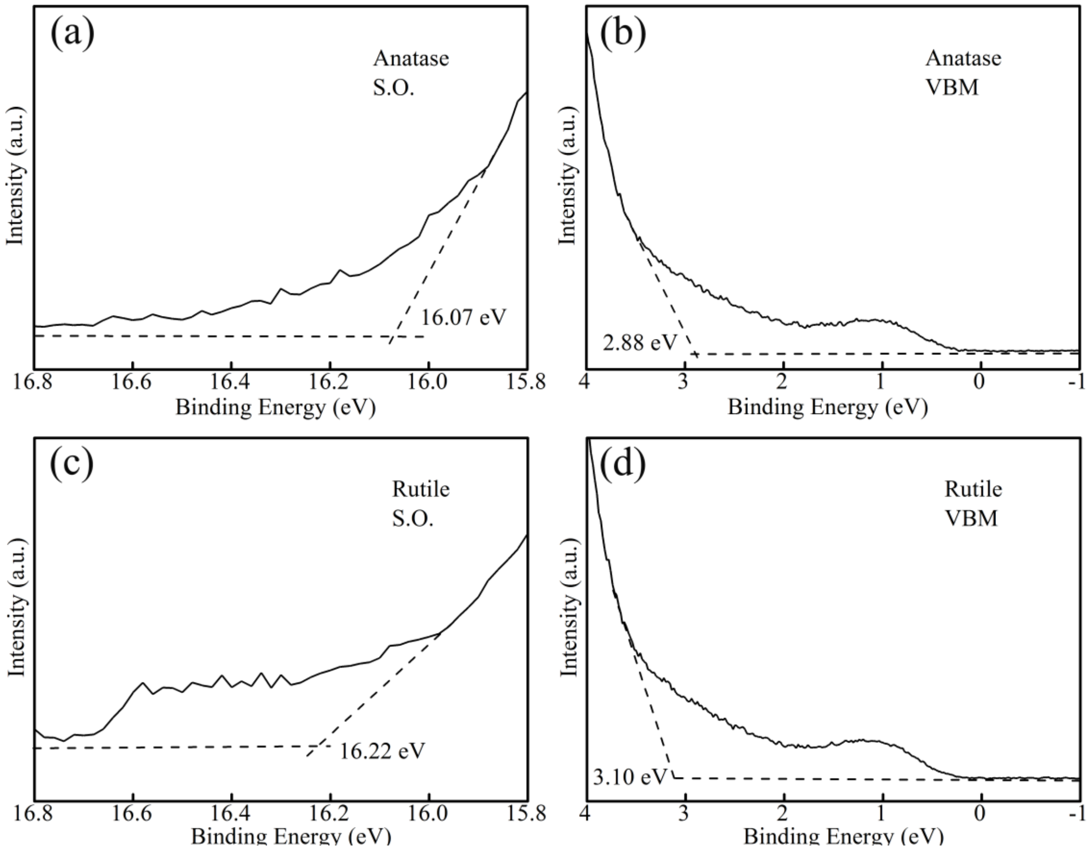

Figure S4. a-d) UPS spectrum of anatase and rutile TiO_2, showing a, c) the secondary electron onset and b, d) the valence band maximum.

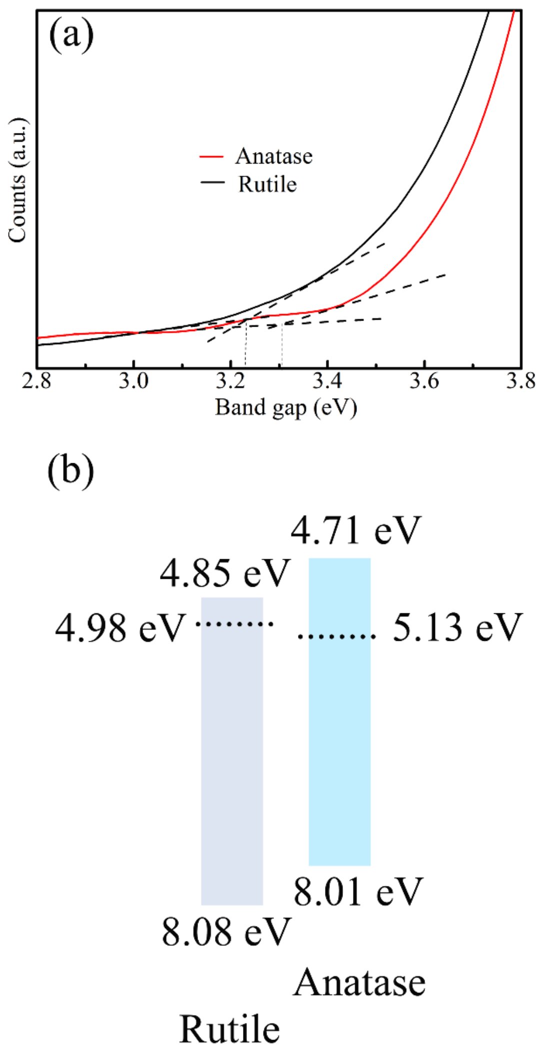

Figure S5. a) The absorption spectra of anatase and rutile TiO_2 films and their fitted bandgap. b) Band alignment between the rutile and anatase films.

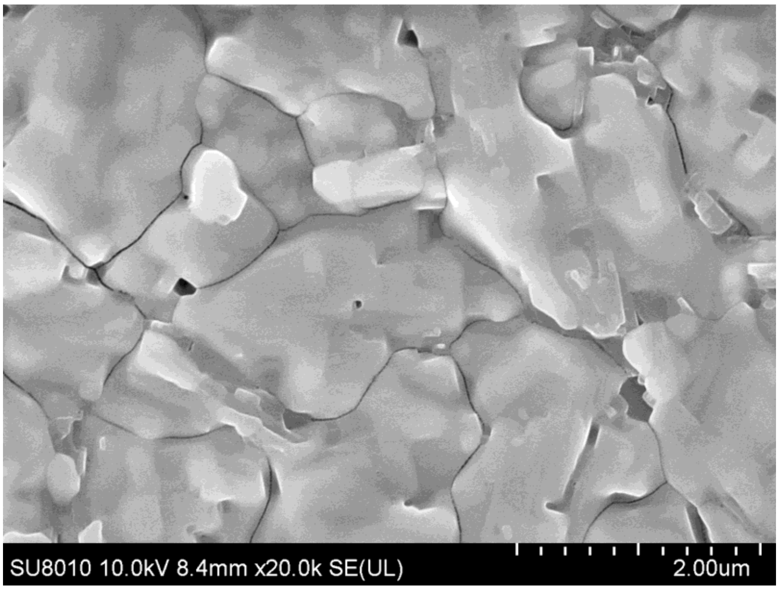

Figure S6. Top-view SEM images of perovskite film on 10 nm-AR TiO_2/FTO substrates.

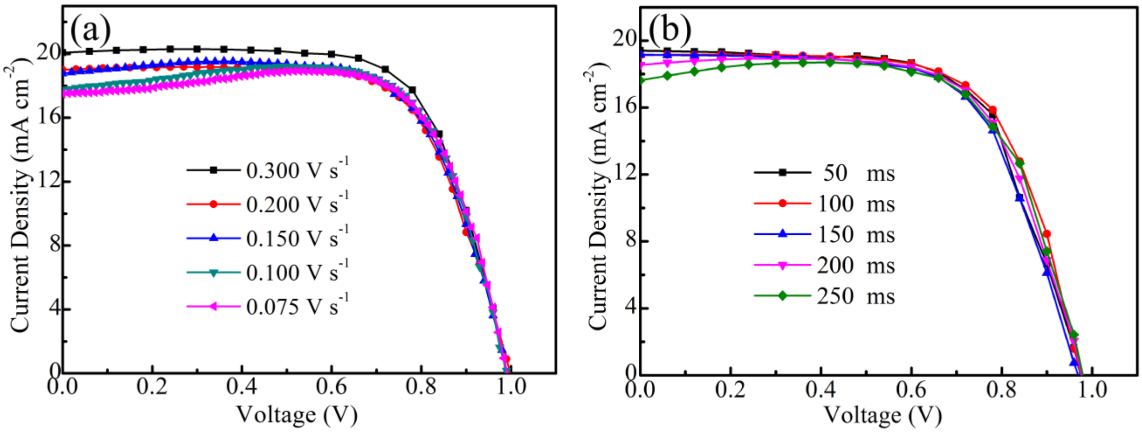

Figure S7. *J*-*V* curves of RA-25 nm devices with different a) scan rates and b) waiting time.

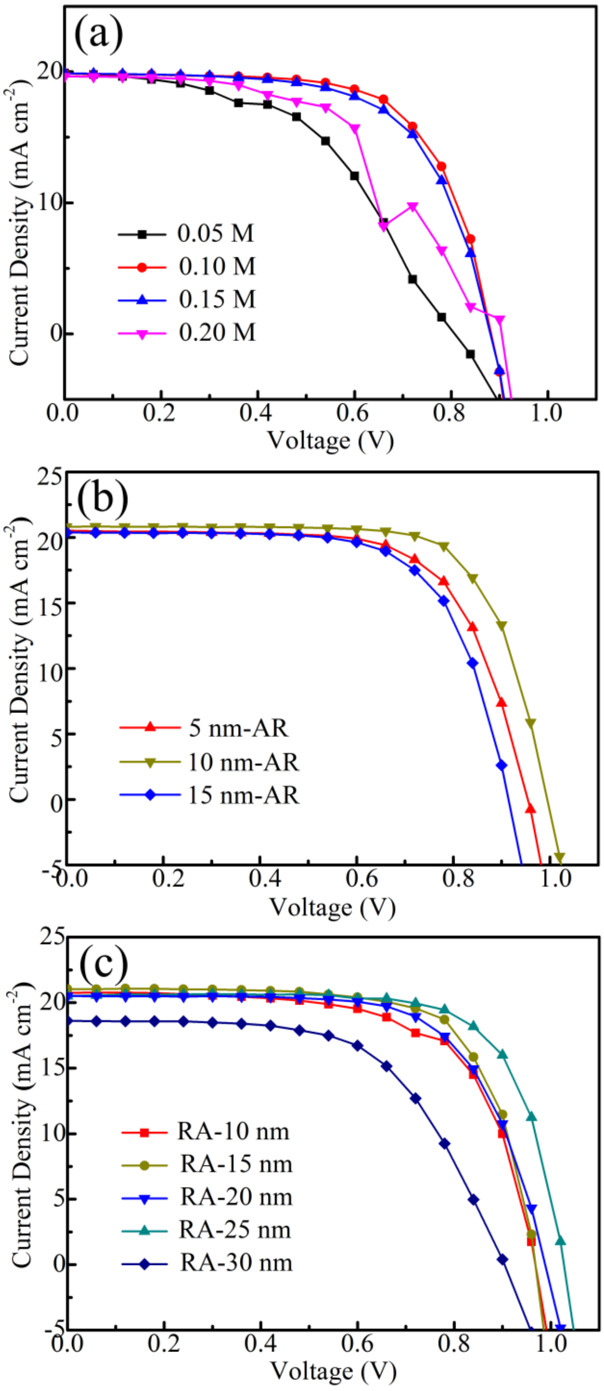

Figure S8. a) *J-V* curves of PSCs based on a) rutile ETLs synthesized under different $TiCl_4$ concentrations, b) AR and c) RA ETLs with different thicknesses of ALD TiO_2.

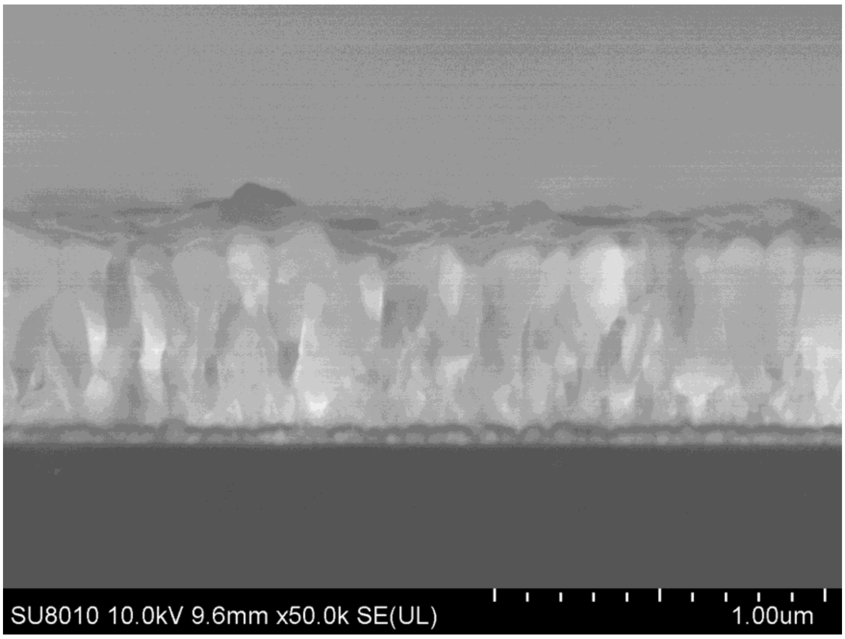

Figure S9. The cross-sectional SEM image of rutile TiO_2 layer from $TiCl_4$ solution.

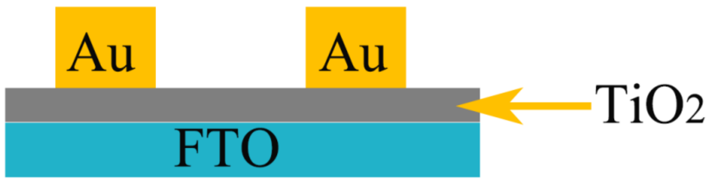

Figure S10. Schematic of hole-only devices.

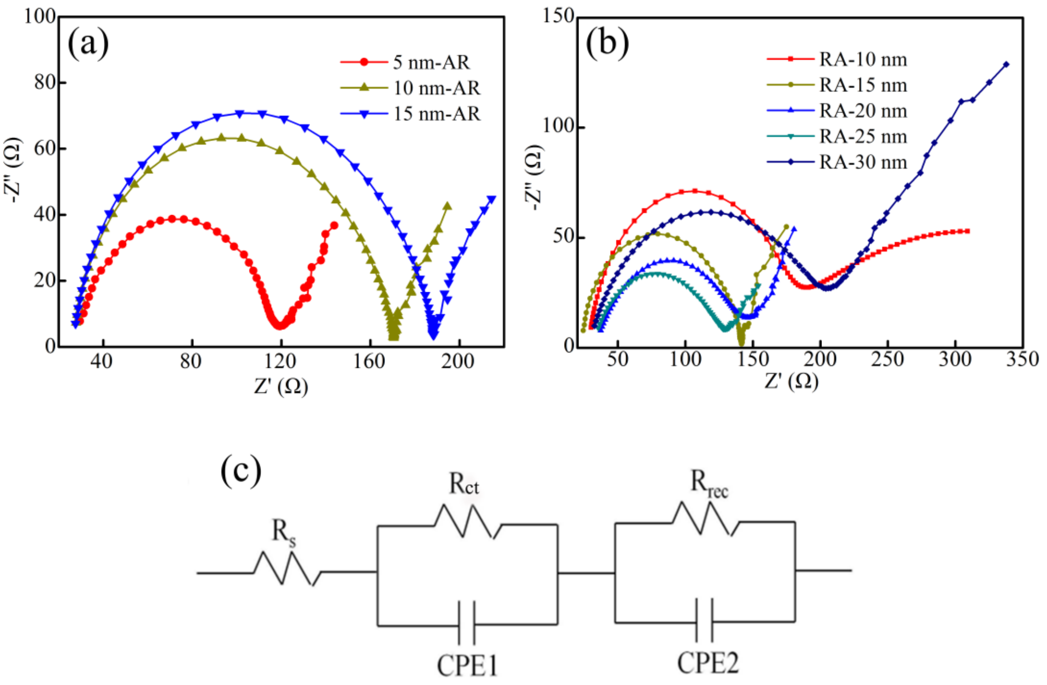

Figure S11. Nyquist plots of devices based on a) AR and b) RA ETLs. c) Equivalent circuit model.

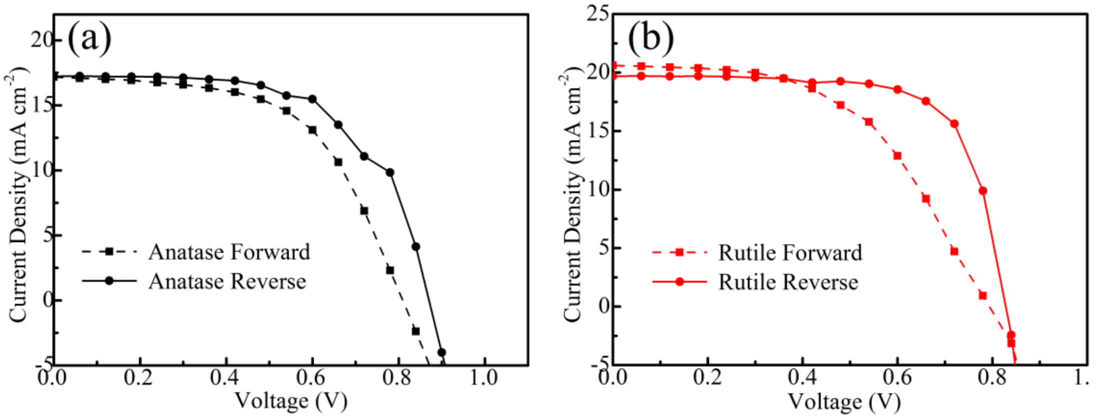

Figure S12. The *J*-*V* curves measured in the forward scan (FS) and reverse scan (RS) direction. a) Anatase and b) rutile.

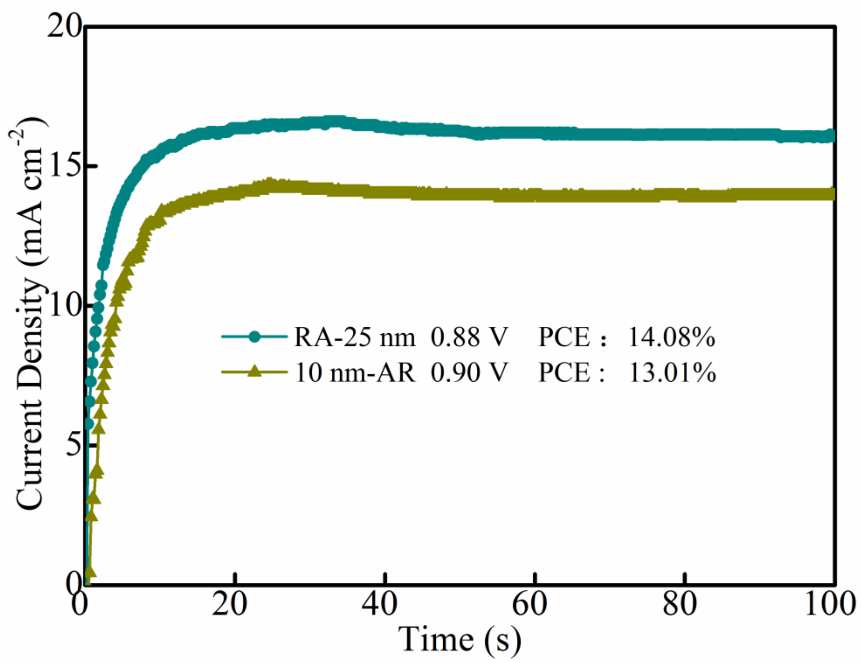

Figure 13. Output current densities of 10 nm-AR and RA-25 nm devices measured at the maximum power point for 100 s.

Devices	R_s (Ω)	R_{ct} (Ω)	R_{rec} (Ω)
Anatase	27.5	157	356
5 nm-AR	28.8	105	557
10 nm-AR	29.1	87	571
15 nm-AR	26.7	131	560
Rutile	32.6	110	292
RA-10 nm	27.1	134	301
RA-15 nm	24.3	114	498
RA-20 nm	34.7	108	754
RA-25 nm	30.5	95	766
RA-30 nm	28.8	163	646

Table S1 The fitted EIS values of PSCs.

Devices	V_{oc} (V)	J_{sc} (mA cm^{-2})	FF (%)	PCE (%)
10 nm-AR (reverse)	0.99	20.83	67.72	14.04
10 nm-AR (forward)	0.88	20.90	55.14	10.12
RA-25 nm (reverse)	0.97	20.32	70.65	13.90
RA-25 nm (forward)	0.93	19.36	66.50	12.05
Anatase (reverse)	0.87	17.23	62.14	9.32
Anatase (forward)	0.81	17.18	57.12	7.94
Rutile (reverse)	0.82	19.70	71.09	11.60
Rutile (forward)	0.79	20.61	52.27	8.55

Table S2 The photovoltaic parameters of devices.
